# The Evolution of Modern Ablative Surgery for the Treatment of Obsessive-Compulsive and Major Depression Disorders

**DOI:** 10.3389/fnint.2022.797533

**Published:** 2022-04-06

**Authors:** Martina Laetitia Mustroph, G. Rees Cosgrove, Ziv M. Williams

**Affiliations:** ^1^Department of Neurosurgery, Brigham and Women’s Hospital, Harvard Medical School, Boston, MA, United States; ^2^Department of Neurosurgery, Massachusetts General Hospital, Harvard Medical School, Boston, MA, United States; ^3^Harvard-MIT Division of Health Sciences and Technology, Boston, MA, United States; ^4^Program in Neuroscience, Harvard Medical School, Boston, MA, United States

**Keywords:** cingulotomy, subcaudate tractotomy, psychosurgery, OCD, depression, limbic leucotomy

## Abstract

In this review, we describe the evolution of modern ablative surgery for intractable psychiatric disease, from the original image-guided cingulotomy procedure described by Ballantine, to the current bilateral anterior cingulotomy using MRI-guided stereotactic techniques. Extension of the single lesion bilateral cingulotomy to the extended bilateral cingulotomy and subsequent staged limbic leucotomy (LL) is also discussed. Other ablative surgeries for psychiatric disease including subcaudate tractotomy (SCT) and anterior capsulotomy (AC) using modern MRI-guided ablative techniques, as well as radiosurgical capsulotomy, are described. Finally, the potential emerging role of MR-guided focused ultrasound (MRgFUS) for treating conditions such as major depressive disorder (MDD) and obsessive-compulsive disorder (OCD) is discussed.

## Introduction

Surgery for psychiatric illness and behavioral disorders has been performed since ancient times. In 1936, Egas Moniz published his first series of 20 patients who underwent prefrontal leucotomies for severe depression, anxiety, and aggression, performed by free-hand injections of absolute alcohol into the frontal lobes, resulting in substantial symptom improvement in 14 of 20 patients (Moniz, 1936a,b; Staudt et al., 2019). Moniz later described his experience with larger numbers of patients after creating more specific and restricted frontal lobe lesions using a hand-held leucotome in lieu of alcohol injections, again with reported overall improvement in the majority of patients (Moniz, 1937; Staudt et al., 2019). These early experiences resulted in Moniz receiving the Nobel Prize in Medicine in 1949 and his coining of the term “psychosurgery” (Feldman and Goodrich, 2001). However, diagnostic and evaluation criteria differed in this era, and while successes with Moniz’s techniques were reported, questions remain regarding possible reporting bias and inaccurately measured responses. In addition, the era lacked the rigor of reporting adverse events that is customary in modern psychosurgery. For instance, in his 1937 paper, Moniz states “limbic leucotomy is a simple operation, always safe,” without citing a single adverse event (Moniz, 1937).

Freeman and Watts (1937) subsequently described their prefrontal lobotomy, which was performed free-hand with a specially designed, calibrated instrument that was inserted blindly to the midline and swept back and forth to surgically interrupt frontal lobe white matter tracts. The authors noted that in order to achieve the best results, the lesion had to involve the medial frontal lobes and the cingulate gyri (Freeman and Watts, 1937). Freeman, a neurologist, later refined the procedure into the transorbital leucotomy, the infamous “ice-pick procedure” (Pressman, 1988; Staudt et al., 2019). The transorbital leucotomy then became widely practiced as Freeman traveled across the United States publicizing and performing his procedure (Staudt et al., 2019). Subsequently, William Scoville, a neurosurgeon, developed the open but more selective orbital undercutting technique through bifrontal trephines (Scoville, 1949; Staudt et al., 2019).

These early operations for psychiatric disease were associated with significant morbidity and mortality. In their review of 10,365 prefrontal lobotomy operations performed between 1943 and 1954, Tooth and Newton confirmed the 70% rate of improvement, but also found a mortality rate of 6%, a rate of post-procedure new-onset epilepsy of 1%, and post-procedure marked disinhibition in 1.5% of patients (Scoville, 1949; Staudt et al., 2019). There were no effective medical therapies for intractable psychiatric disease until 1950, when chlorpromazine became available. In this context, complications from early operations for psychiatric disease were considered acceptable but underscored the need for less radical and more refined approaches to such surgeries.

## Early Stereotactic Ablative Surgery

**FIGURE 1:**
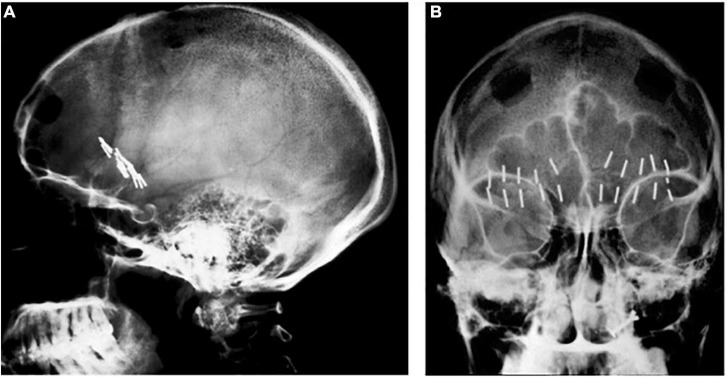
Knight’s early subcaudate tractotomy. **(A)** Lateral and **(B)** AP X-rays showing bilateral yttrium seed implants.

**FIGURE 2:**
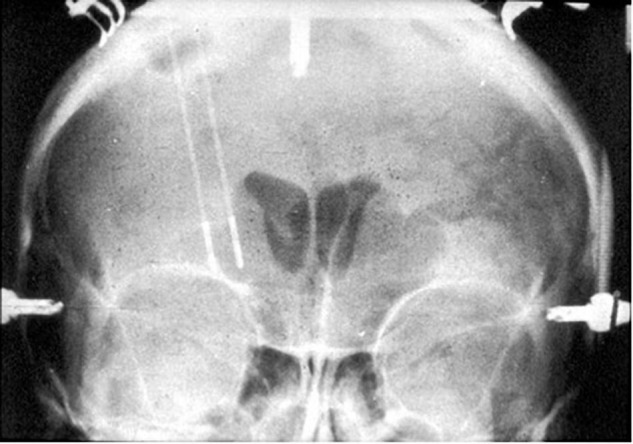
Coronal X-ray of electrodes in place for creation of Leksell’s right frontal capsulotomy lesions.

A major milestone in precision targeting for ablative surgery for psychiatric disease occurred in 1947, when Spiegel and Wycis introduced human stereotaxy as a method to more reproducibly and safely perform psychiatric surgery (Spiegel et al., 1947). Spiegel and Wycis employed X-ray ventriculography to target the dorsomedial nucleus of the thalamus with the goal of reducing emotional reactivity (Spiegel et al., 1947; Staudt et al., 2019), which represents the first use of image guidance using lateral and AP X-rays in psychosurgery. In 1965, Geoffrey Knight adapted Scoville’s orbitofrontal undercutting procedure and pioneered the stereotactic subcaudate tractotomy (Knight, 1965; Staudt et al., 2019). Using the pituitary sella as a landmark, Knight inserted rod-shaped yttrium seeds bilaterally into the posterior orbito-frontal cortex to create radiation necrotic lesions (Lapidus et al., 2013; Staudt et al., 2019; Figure 1). Knight later employed radiofrequency coagulation to generate lesions (Lapidus et al., 2013; Staudt et al., 2019). In 1949, Jean Talairach first described the bilateral anterior capsulotomy for psychiatric disease (anxiety neuroses and obsessive compulsive disorders), for which he used thermocoagulation (Talairach et al., 1949; Spiegelmann et al., 1994; Zanello et al., 2017; Harary and Cosgrove, 2019). He later modified his technique from using thermocoagulation to implanting radioactive yttrium-90 seeds with a 2 mm needle (Zanello et al., 2017). Independently from Talairach in 1950, Lars Leksell used an air ventriculogram and parallel bipolar radiofrequency electrodes to lesion the anterior limb of the internal capsule (Leksell, 1950; Figure 2).

Open cingulotomy procedures were performed prior to Ballantine’s 1962 pioneering stereotactic cingulotomy procedure. In 1952, Sir Hugh Cairns published a description of an open cingulotomy procedure undertaken between 1948 and 1951 in 29 patients for anxiety, obsessions, psychosis, depression, postencephalitic behavior disorder, mania, and schizophrenia (Whitty et al., 1952; Staudt et al., 2019). For the open cingulotomy, Cairns used a unilateral right frontal approach with-if necessary- division of the lower falx to expose and then remove via suction the bilateral anterior half of the cingulate gyri, which reportedly measured 4 cm in length × 1 cm in depth × 1 cm in width, thereby undertaking a subtotal resection of Brodmann area 24 (Whitty et al., 1952). In 24 of 29 patients, Cairns divided venous branches of the superior sagittal sinus to retract the medial right frontal lobe surface from the falx and reported “on a few occasions” small subpial extravasation of blood in gyri. Cairns conceded that his technique probably produced diffuse damage to the frontal lobes and championed preserving as many draining veins as possible (Whitty et al., 1952). Three of Cairns’ open cingulotomy patients experienced new-onset seizures after the procedure (Whitty et al., 1952). Cairns also reported one death due to infection; in the remaining 28 cases, at follow-up ranging from 18 months to 3 years, Cairns reported very limited personality changes but robust changes in mental illness in most patients, noting that in patients with psychosis the improvement from surgery was only transient (Whitty et al., 1952). The open cingulotomy procedure was unsuccessful in the one patient in whom only a portion of the unilateral anterior cingulate gyrus of the dominant hemisphere was removed (Whitty et al., 1952). Symptoms in the same patient improved 3 months after reoperation in which the anterior cingulate gyrus of the non-dominant hemisphere was removed (Whitty et al., 1952).

**FIGURE 3:**
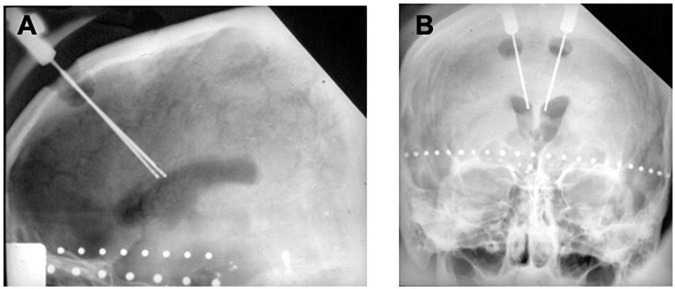
Original ballantine cingulotomy. **(A)** Lateral and **(B)** AP X-ray of electrodes in place for creation of a cingulotomy lesion.

In 1962, Ballantine performed the first stereotactic bilateral cingulotomy, aided by an air ventriculogram and X-rays (Ballantine et al., 1967; Dyster et al., 2016). For this procedure, Ballantine made a bifrontal incision, followed by bilateral burr holes 1.5 cm lateral from midline and 9 cm posterior to nasion (Ballantine et al., 1967). He opened the dura in a cruciate fashion, lightly cauterized the underlying cortex, and then sutured the scalp incision closed. After 5 cc of air were injected into each lateral ventricle, custom-designed ventricular needles that were electrically insulated except for their distal 1 cm tips were manually introduced through the closed scalp incision. Using lateral and AP X-rays for image guidance and a 1 cm beaded tape measurer for scale (Ballantine et al., 1967), Ballantine positioned the needles in the cingulate gyrus 1 cm lateral to midline and 2.5 cm posterior to the frontal horn of each lateral ventricle and then used the needles as lesion-making electrodes with a monopolar radiofrequency current (Ballantine et al., 1967; Brotis et al., 2009; Figure 3). Between 1962 and 1966, Ballantine made several adjustments to his operative approach. Ballantine increased the amount of current from 2 to 8 watts for a duration of 15–30 s to a larger current and longer duration of 8 watts for 60 s in order to expand lesion size and preempt the need for reoperation (Ballantine et al., 1967). Ballantine subsequently adjusted his technique and would create a second, more dorsally located lesion by withdrawing the electrode 1 cm and running current a second time, resulting in bilateral lesions of approximately 2 cubic cm each (Ballantine et al., 1967).

In Ballantine’s original series of patients with neuropsychiatric illness (*n* = 57) or intractable chronic pain from incurable cancer (*n* = 12) who underwent cingulotomy, 79% of patients showed symptom improvement, an early finding heralding the utility of ablative surgery for psychiatric disease (Ballantine et al., 1967). In a later study with longer follow-up, Ballantine found that 13% of 198 patients with intractable psychiatric disease who underwent ablative cingulotomy fully recovered, from their psychiatric disease and 23% returned to normal functioning, albeit under continued psychiatric care (Ballantine et al., 1987). Affective disorders responded most favorably to bilateral stereotactic cingulotomy, with unipolar, bipolar, and schizoaffective disorder subtypes responding equally well; 64% (77 of 120) patients with affective disorder who underwent cingulotomy recovered or showed marked improvement (Ballantine et al., 1987). A reported 56% of patients with OCD who underwent cingulotomy recovered or showed marked improvement (Ballantine et al., 1987). Re-analysis of Ballantine’s data using more rigid criteria and clinically validated rating scales for OCD found this figure to be lower, or 33% (Cosgrove, 2000; Patel et al., 2013).

## Modern Stereotactic Ablative Surgery

**FIGURE 4:**
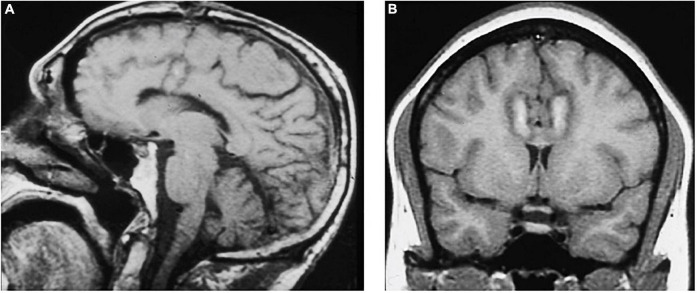
MRI-guided cingulotomy. **(A)** Sagittal and **(B)** Coronal T1-weighted MRI of acute single lesion cingulotomy.

The modern cingulotomy procedure underwent several refinements until it reached its current form. With the advent of more advanced imaging technologies, the cingulotomy procedure evolved into a stereotactic frame-based procedure using at first CT and then MRI-guidance for targeting. In keeping with Ballantine’s targeting methods, MRI-guided cingulotomy used sagittal T1-weighted images to identify the cingulate gyri bilaterally and to approximate the location of burr holes and planned electrode trajectories. Oblique coronal images were then obtained to calculate target coordinates for a point in the anterior cingulate gyrus 2–2.5 cm posterior to the tip of the frontal horns, 7 mm lateral from midline, and 1 mm superior to the roof of the ventricles bilaterally (Spangler et al., 1996). A 10 mm electrode with an uninsulated tip was lowered to the target coordinates and heated to 85°C for 60 s to make a radiofrequency thermocoagulation lesion. The electrode was then withdrawn 10 mm, and a second lesion was made, resulting in a final cingulate lesion approximately 2 cm in height and 8–10 mm in diameter (Spangler et al., 1996; Figure 4).

**FIGURE 5:**
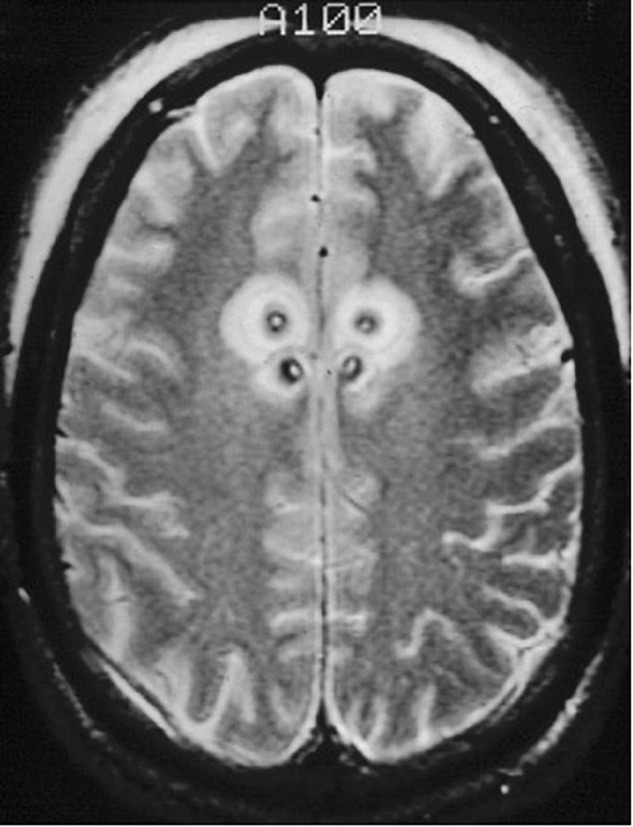
Staged cingulotomy. Transverse T2-weighted MRI of chronic (posterior) and acute (anterior) double cingulotomy lesions.

**FIGURE 6:**
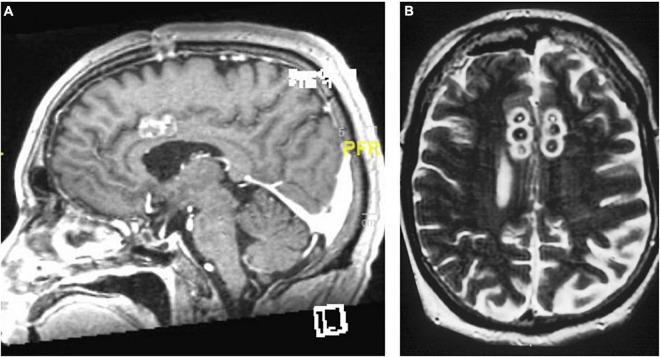
The “six-pack” cingulotomy. **(A)** Sagittal and **(B)** Axial T2-weighted MRI of chronic triple lesion cingulotomy.

Performed under local anesthesia with intravenous sedation using a CRW frame, this standard bilateral anterior cingulotomy with a single 2 cm × 1 cm lesion was the initial intervention for patients from 1986 until 1996 (Shields et al., 2008). Patients who were considered non-responders or showed only minimal response after 9–12 months underwent additional surgery, with extension of the cingulate lesion anteriorly (Figure 5). Patients who remained non-responders after the second cingulotomy often underwent a third cingulotomy. Overall response rates improved with each additional procedure. To reduce the risks and expense of multiple procedures, in 1996 the decision was made to create all three anterior cingulotomy lesions simultaneously (Shields et al., 2008). The triple lesion cingulotomy or so-called “six-pack” procedure, in which three cingulate lesions on each side are made, became the standard cingulotomy after 1996. The “six-pack” cingulotomy lesions are made through a single burr hole using a 10 mm exposed tip electrode by re-directing the electrode trajectory after the first lesion and adjusting to the contour of the cingulate gyrus (Shields et al., 2008; Schweder and Cosgrove, 2012; Figure 6).

In a cohort of 64 patients with medically refractory OCD who underwent stereotactic MRI-guided cingulotomy, 42% of patients were full or partial responders at 11-month follow-up, a percentage that increased to 69% by 64-month follow-up (Sheth et al., 2013). Thirty of the study’s 64 patients underwent a subsequent procedure after initial anterior cingulotomy – either repeat anterior cingulotomy or – if they had already undergone a triple cingulotomy – subcaudate tractotomy (Sheth et al., 2013). A study of 31 patients with severe treatment-refractory OCD who remained non-responders after anterior cingulotomy but achieved a higher response rate after subcaudate tractotomy showed that the subcaudate tractotomy might confer a higher response rate than repeat cingulotomy (Bourne et al., 2013).

## Limbic Leucotomy

In the mid-1990’s at Massachusetts General Hospital, in instances in which patients failed to respond to the initial “six-pack” cingulotomy, patients were offered a subcaudate tractotomy. The combination of cingulotomy followed by subcaudate tractotomy resulted in a staged limbic leucotomy. Kelly et al. (1973) first described the original limbic leucotomy procedure. He reasoned that if cingulotomy and subcaudate tractotomy independently led to symptom benefit, then combining the procedures might confer even greater improvement and better results overall. In a series of 66 patients with intractable psychiatric disease who underwent limbic leucotomy, Kelly reported improvement in 89% of patients with OCD and in 78% of patients with MDD (Kelly et al., 1973; Montoya et al., 2002). The initial success of this staged limbic leucotomy procedure formed the basis for performing a limbic leucotomy on some of the most severely affected patients as the initial procedure.

**FIGURE 7:**
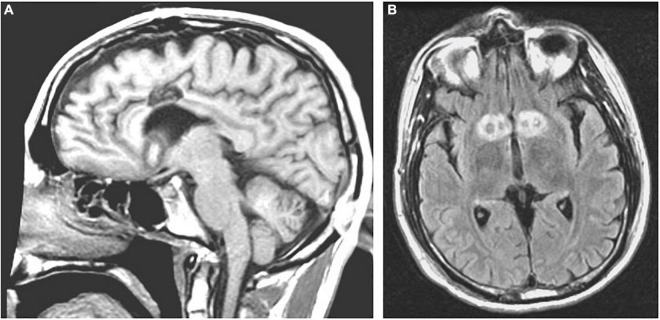
Limbic leucotomy. **(A)** Sagittal T1-weighted and **(B)** Axial FLAIR MRI of limbic leucotomy lesions.

The modern limbic leucotomy is performed through bilateral burr holes, beginning with a single anterior cingulotomy lesion using a 10 mm exposed electrode at the aforementioned coordinates. Targeting for the subcaudate tractotomy lesion in the posteroinferomedial quadrant of the frontal orbital cortex begins just above the floor of the anterior cranial fossa, 7 mm lateral to midline and 12–15 mm anterior to the anterior commissure. The same 10 mm exposed electrode is inserted and heated to 85°C for 60 s and then withdrawn 5 mm and heated again to create a lesion 15 mm in height and 10 mm in diameter. The medial lesion extends into subcallosal cingulate gyrus (CG 25). A second lesion is placed 14 mm from the midline on the same plane using similar parameters. The upper portion of this lesion should extend into the inferior portion of the anterior limb of the internal capsule (Montoya et al., 2002; Figure 7).

Limbic leucotomy is an effective treatment for both MDD and OCD; in a study of 21 patients with chronic, severe OCD (71%) or MDD (29%) who underwent MRI-guided stereotactic limbic leucotomy either as a first, second, or third procedure, 54% of MDD patients and 40% of OCD patients responded fully or partially at 46-month follow-up, with full response defined as a 50% reduction on the Beck Depression Inventory (BDI) score (for MDD patients) or a 35% reduction on the Yale-Brown Obsessive-Compulsive Scale (YBOCS) score (for OCD patients). Postoperative sequelae of apathy, seizures, urinary incontinence, and memory problems occurred in a minority of patients (Montoya et al., 2002). Interestingly, limbic leucotomy was effective in only 20% of patients when performed as the initial procedure, but in 50% of patients when performed as a staged procedure after one or multiple failed cingulotomies. The greater benefit conferred by staged as opposed to initial limbic leucotomy may reflect either the greater illness severity of patients who tend to be selected for an upfront limbic leucotomy procedure or the longer follow-up period between initial procedure and follow-up in patients who undergo staged procedures (Montoya et al., 2002). Limbic leucotomy has also been shown to significantly reduce severe self-mutilation behavior in patients with intractable OCD or schizoaffective disorder who are otherwise unresponsive to interventions (Price et al., 2001).

## Radiofrequency Capsulotomy

Capsulotomy can be performed using either of two stereotactic techniques: radiofrequency heating (thermocapsulotomy) or gamma radiation (radiosurgical capsulotomy or gamma capsulotomy) (Lapidus et al., 2013; Brown et al., 2016). Radiofrequency capsulotomy is effective at reducing treatment-refractory OCD (Oliver et al., 2003; Liu et al., 2008; Rück et al., 2008; Csigó et al., 2010) and anxiety symptoms (Rück et al., 2003) but has been associated with significant adverse effects, including executive dysfunction, sexual disinhibition, apathy, urinary incontinence, and weight gain (Oliver et al., 2003; Rück et al., 2003, 2008; Csigó et al., 2010), in addition to isolated reports of self-limited seizure activity and transient hallucinations (Oliver et al., 2003). Adverse effects of radiofrequency capsulotomy include at least one reported suicide and one reported case of a patient who became severely sexually disinhibited immediately after surgery and was convicted of rape 5 months after surgery (Rück et al., 2008). A non-randomized comparison of 25 patients with treatment-refractory OCD at the Karolinska Institute who underwent either radiofrequency or radiosurgical capsulotomy between 1988 and 2000 suggests that radiofrequency capsulotomy is associated with greater adverse effects than radiosurgical capsulotomy (Rück et al., 2008).

## Radiosurgical Capsulotomy

**FIGURE 8:**
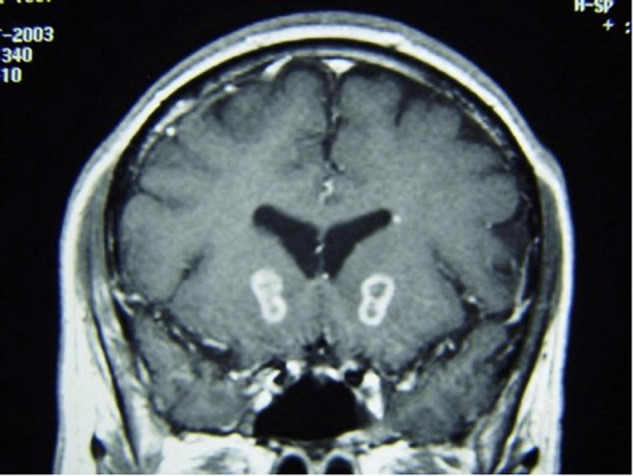
Coronal T1-weighted MRI with contrast of gamma knife capsulotomy lesions.

Essentially the entirety of the stereotactic radiosurgery (SRS) capsulotomy experience utilized the Gamma Knife (see Miguel et al., 2019 for a much more detailed account of the evolution of the Gamma Knife capsulotomy). Capsulotomy was initially performed with radiofrequency probes. Leksell developed the Gamma Knife as a non-invasive radiosurgical alternative to performing stereotactic capsulotomy for psychiatric illness (Leksell, 1987; Lindquist and Kihlström, 1996; Chodakiewitz et al., 2015). In Gamma Knife capsulotomy, radiation doses of 140–180 Gy, narrowed by 4 mm collimaters, are targeted to converge at an isocenter in the brain (Leksell, 1987; Lindquist and Kihlström, 1996; Chodakiewitz et al., 2015). Initial targets were chosen to treat so-called “functional disorders,” such as intractable pain, trigeminal neuralgia, parkinsonism, epilepsy, and psychoneurosis, including severe anxiety and OCD (Leksell, 1987). Between 1968 and 1986, 1,311 capsulotomy procedures were performed at the Karolinska Institute (Leksell, 1987). The initial experience of Gamma Knife capsulotomy in OCD patients from the Karolinska Institute suggested that radiosurgical capsulotomy is as effective as thermocapsulotomy (Lippitz et al., 1999). Criteria of good postoperative outcome were met by 9 of 19 patients who underwent bilateral thermocapsulotomy for OCD and by 7 of 10 patients who underwent radiosurgical capsulotomy for OCD (Lippitz et al., 1999). T2-weighted MRI of stereotactic lesions in the internal capsule can accurately assess lesion location (Mindus et al., 1986). Lesions in specific areas of the right internal capsule after radiosurgery for OCD correlate with symptom improvement (Lippitz et al., 1997; Figure 8).

In an early trial of radiosurgical capsulotomy for OCD at Brown University in which a single lesion using 140 Gy was made, only 1 of 15 patients met criteria for full or partial responder at 9-month follow-up (Rasmussen et al., 2018). Thirteen of these 15 patients opted to undergo a second, more ventral Gamma Knife capsulotomy, after which 7 of the 13 patients who had been previously unresponsive became responders (Rasmussen et al., 2018). As a group, these “double shot” patients maintained significant symptom improvement at 12-, 24-, and 36-month follow-up (Rasmussen et al., 2018). A subsequent trial of “double shot” Gamma Knife capsulotomy for OCD in 40 patients found significant OCD symptom improvement at 6-month follow-up, an effect that persisted at 12-, 24-, and 36-month follow-up (Rasmussen et al., 2018). No lasting change in normal neuropsychological performance was observed in any patient. Other centers observed similar results of radiosurgical capsulotomy for refractory OCD in smaller series of 10 and 20 patients; 70-75% of patients were full responders, experiencing at least a greater than 35% reduction in YBOCS scores at 5-year follow-up (Spatola et al., 2018; Peker et al., 2020). Radiosurgical capsulotomy reduced obsessions, compulsions, depression, and anxiety, and markedly improved quality of life (Spatola et al., 2018). A prospective randomized clinical trial of radiosurgical ventral capsulotomy for OCD in which two distinct isocenters were created on each side in eight patients did not achieve statistical significance, although 3 of 8 treated patients were considered responders (Lopes et al., 2014, 2015; Baethge, 2015).

**FIGURE 9:**
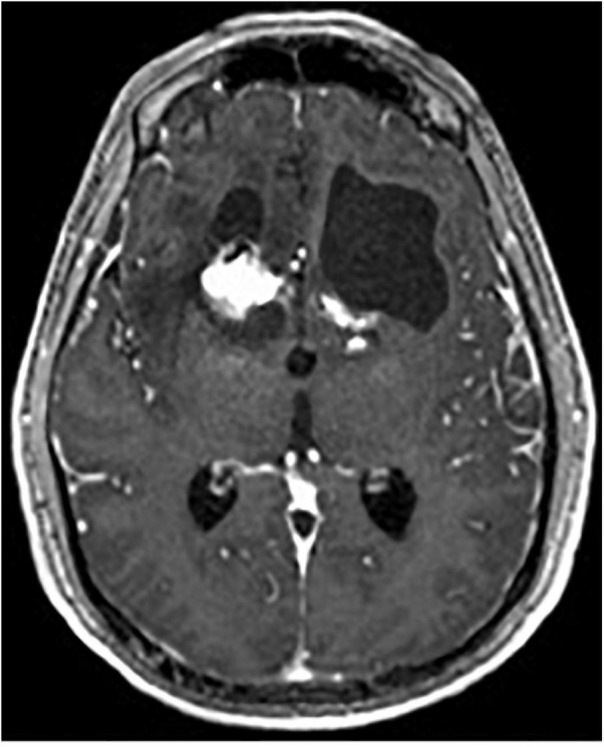
Axial T1-weighted MRI with contrast of brain cyst in the left hemisphere after gamma knife capsulotomy.

One complication of radiosurgical capsulotomy is the development of brain cysts, which have been found in 10% of patients at 5-year follow-up (Peker et al., 2020). They appear to be a radiation dose-dependent phenomenon, with doses above 140 Gy predicting occurrence, whereas lower doses of 110 Gy appear to be safe (Kihlström et al., 1997; Kasabkojian et al., 2021; Figure 9).

## Laser Interstitial Thermal Therapy

**FIGURE 10:**
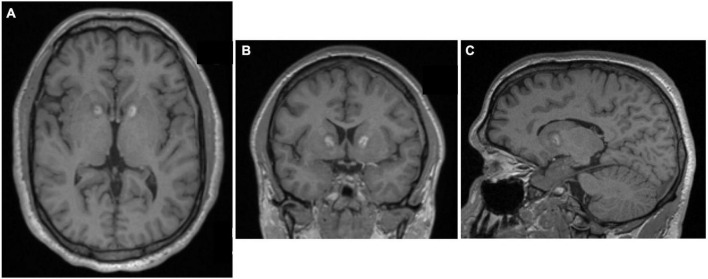
Laser interstitial thermal therapy (LITT) capsulotomy for OCD. **(A)** Axial, **(B)** Coronal, and **(C)** Sagittal MPRAGE MRI images after MR-guided LITT. Original figures kindly provided by Dr. Wael F. Asaad, Brown University.

The delayed development of brain cysts after radiosurgical capsulotomy prompted the Brown group to employ an alternative lesioning approach: MRI-guided laser interstitial thermal therapy (LITT) (McLaughlin et al., 2021). In the largest series of LITT for the treatment of psychiatric disease to date, 77.8% of ten patients with severe, intractable OCD who underwent MR-guided LITT capsulotomy were full responders, with full response defined as at least a 35% reduction in YBOCS scores (McLaughlin et al., 2021). The procedure was generally well tolerated, and it appears to have a comparable efficacy to radiosurgical capsulotomy (McLaughlin et al., 2021). Overall responses to LITT are comparable to SRS, but interval to symptom response was much shorter are LITT (Figure 10).

## MR-Guided Focused Ultrasound

**FIGURE 11:**
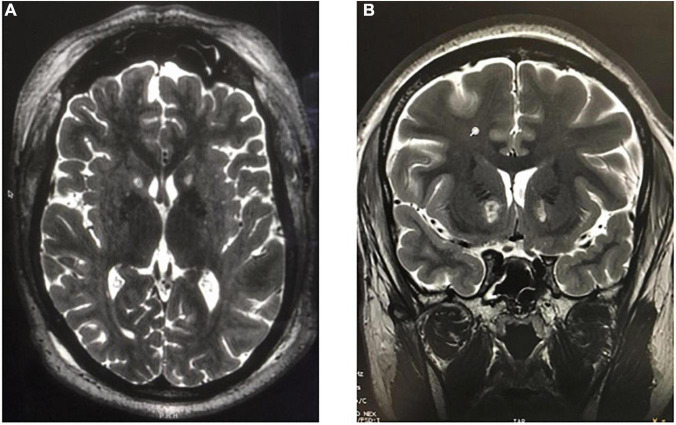
MR-guided focused ultrasound capsulotomies (MRgFUS). **(A)** Axial T2-weighted and **(B)** Coronal T2-weighted MRI after bilateral MRgFUS capsulotomy.

Another recently developed non-invasive lesioning technique for refractory OCD or MDD is MR-guided focused ultrasound (MRgFUS). In this procedure, a stereotactic head frame is applied with local anesthesia to the patient’s shaved head and secured in a commercially manufactured helmet embedded with 1,024 ultrasound transducers (ExAblate Neuro; InSightec Inc., Haifa, Israel) within the MRI scanner. In MRgFUS for OCD or MDD, the anterior capsule is directly targeted, and the target area is heated to 50–60°C for several seconds under real-time MR-thermometry monitoring (Davidson et al., 2020a; Figure 11).

A proof-of-concept study of MRgFUS anterior capsulotomy in 4 patients with treatment-resistant MDD found significant symptom relief in 3 of 4 patients within 1 week, and at least a 50% reduction in hamilton depression rating scale (HDRS) scores in all patients at 12-month follow-up (Jung et al., 2015). A follow-up study of 11 patients who underwent MRgFUS anterior capsulotomy for medically refractory OCD showed significant reduction in YBOCS and HDRS scores at 2 years (Kim et al., 2018). In 16 patients who underwent MRgFUS capsulotomy for MDD or OCD, no serious adverse events occurred, although in six additional patients, lesions could not be made due to limiting skull densities (Davidson et al., 2020a). At 6-month follow-up, six of the 12 patients who had undergone MRgFUS capsulotomy and reached 6-month follow-up showed clinical response, defined as a reduction of at least 35% on the YBOCS score (for patients with OCD) or a reduction of at least 50% on the Hamilton Depression Rating Scale (HDRS) (for patients with MDD) (Davidson et al., 2020a). Two phase I trials of MRgFUS anterior capsulotomy for MDD (*n* = 6) and OCD (*n* = 6) found response rates of 2 of 6 patients with OCD and 4 of 6 patients with MDD, respectively (Davidson et al., 2020b). Overall response rate and magnitude of response to MRgFUS capsulotomy are comparable to SRS capsulotomy, but the interval to response is much shorter with MRgFUS. Going forward, diffusion tensor imaging (DTI) may be used to achieve more patient-specific targeting of the tracts in the anterior limb of the internal capsule for capsulotomies, with the potential for even better outcomes (Avecillas-Chasin et al., 2019).

## Discussion

The burden of psychiatric disease in our society remains extremely high (Steketee, 1997; Greenberg et al., 2003, 2015; Hankin et al., 2011; Murray, 2013; Ärnlöv and Larsson, 2015; Cloutier et al., 2016; Wu et al., 2018). Despite modern pharmacological and behavioral therapies, many patients do not respond to existing therapies and remain severely disabled. Some of these patients with psychiatric disease refractory to pharmacological and behavioral therapies are appropriate candidates for surgical intervention. The evolution of modern stereotactic techniques along with novel non-invasive lesioning methods provides additional impetus to reconsider ablative surgical techniques as a reasonable therapeutic option for properly selected patients with severe, disabling and treatment-refractory psychiatric disease.

Neuromodulation therapies such as deep brain stimulation (DBS) and vagus nerve stimulation (VNS) have gained traction due to the fact that they are titratable and reversible, but these non-lesional procedures remain extremely expensive interventions (see Bari et al., 2018; Karas et al., 2018 for more detailed discussion of DBS and VNS for psychiatric disorders). Minimally invasive, incisionless ablative procedures without prohibitive costs are now possible. As a result, there is renewed interest in ablative surgical techniques to treat psychiatric disease. In fact, according to a recent global survey of functional neurosurgeons, over 41% of neurosurgery for psychiatric disease is ablative (Mendelsohn et al., 2013).

The treatment of medically refractory psychiatric disorders with ablative surgery has improved thanks to the development of better techniques, including MRI, MRgFUS, LITT, etc., as outlined in the previous sections. Better understanding of brain function and selection of more suitable targets has led to further improvements in the treatment of psychiatric disorders. For instance, empirically derived surgical targets for OCD with LITT evolved from the bilateral anterior ventral capsule to a target encompassing the bilateral bottom third of the ventral capsule (Rasmussen et al., 2018; McLaughlin et al., 2021). Similarly, treatment of MDD has shifted from the massive lesioning of the medial frontal lobes and cingulate gyri to the refined selective ablation of Cg25 (Holtzheimer et al., 2017). The advent of new technologies such as DTI permits patient-specific targeting of tracts, such as the anterior limb of the internal capsule (Nanda et al., 2017). Complex ethical issues surrounding patient autonomy will continue to require evaluation by a multidisciplinary care team whenever considering ablative surgery for psychiatric disease. We are hopeful that advances in our understanding of the neurocircuitry involved in psychiatric conditions may enhance the efficacy of ablative surgery for medically refractory psychiatric disease in the future.

## Author Contributions

MM and GC jointly outlined the manuscript. MM wrote the manuscript. GC and ZW edited manuscript drafts. All authors contributed to the final manuscript.

## Conflict of Interest

The authors declare that the research was conducted in the absence of any commercial or financial relationships that could be construed as a potential conflict of interest.

## Publisher’s Note

All claims expressed in this article are solely those of the authors and do not necessarily represent those of their affiliated organizations, or those of the publisher, the editors and the reviewers. Any product that may be evaluated in this article, or claim that may be made by its manufacturer, is not guaranteed or endorsed by the publisher.
